# A Review of Cyclist Head Injury, Impact Characteristics and the Implications for Helmet Assessment Methods

**DOI:** 10.1007/s10439-023-03148-7

**Published:** 2023-03-14

**Authors:** Claire E. Baker, Xiancheng Yu, Saian Patel, Mazdak Ghajari

**Affiliations:** HEAD Lab, Dyson School of Design Engineering, Imperial College London, London, SW7 2AZ UK

**Keywords:** Bicycle helmet, Cyclist collision, Head impact, Injury biomechanics, Road traffic collision, Traumatic brain injury

## Abstract

Head injuries are common for cyclists involved in collisions. Such collision scenarios result in a range of injuries, with different head impact speeds, angles, locations, or surfaces. A clear understanding of these collision characteristics is vital to design high fidelity test methods for evaluating the performance of helmets. We review literature detailing real-world cyclist collision scenarios and report on these key characteristics. Our review shows that helmeted cyclists have a considerable reduction in skull fracture and focal brain pathologies compared to non-helmeted cyclists, as well as a reduction in all brain pathologies. The considerable reduction in focal head pathologies is likely to be due to helmet standards mandating thresholds of linear acceleration. The less considerable reduction in diffuse brain injuries is likely to be due to the lack of monitoring head rotation in test methods. We performed a novel meta-analysis of the location of 1809 head impacts from ten studies. Most studies showed that the side and front regions are frequently impacted, with one large, contemporary study highlighting a high proportion of occipital impacts. Helmets frequently had impact locations low down near the rim line. The face is not well protected by most conventional bicycle helmets. Several papers determine head impact speed and angle from in-depth reconstructions and computer simulations. They report head impact speeds from 5 to 16 m/s, with a concentration around 5 to 8 m/s and higher speeds when there was another vehicle involved in the collision. Reported angles range from 10° to 80° to the normal, and are concentrated around 30°–50°. Our review also shows that in nearly 80% of the cases, the head impact is reported to be against a flat surface. This review highlights current gaps in data, and calls for more research and data to better inform improvements in testing methods of standards and rating schemes and raise helmet safety.

## Introduction

Cycling is an increasingly popular recreational activity and green mode of transport. Health benefits and climate awareness additionally provide motivation for people to cycle.^109,117^ One risk associated with cycling is head injury during falls or collisions. Head injuries are a key contributor of fatal and life-changing injuries in cyclists, and helmets are a key line of defence against them.^20^ New helmet technologies have been entering the market at a rapid pace with a range of claims about their head injury mitigation benefits.^1^ There is a need for an independent assessment of cycle helmets to differentiate between these incoming technologies, enabling consumers to make informed choices and manufacturers to better target technology development.

All helmets sold meet regulatory standards. Current standards provide reassurance that a baseline protective threshold is met.^15,32^ However, current standards are outdated as they do not include configurations that best represent real world scenarios. For example, in the EN1078 standard, which is widely adopted across Europe, only normal impacts are prescribed, not oblique impacts.^32^ Oblique impacts, which have both tangential and normal velocity components, have been shown to be more representative of the collisions experienced in the real world.^14,89^ Current standards additionally only include a linear acceleration threshold to assess helmet performance. Linear acceleration is the established injury mechanism for certain head injuries, such as skull fracture and other focal pathologies (such as extradural haematoma secondary to skull fracture).^54,73^ However, both normal and oblique impacts induce head rotation, which is known to be a key mechanism of several distinct types of head injury, such as diffuse axonal injury.^38,39,51,54^ Hence, it is necessary for standards to include rotational head kinematics in addition to linear acceleration. This inclusion must be underpinned by how severely and frequently cyclists sustain injuries caused by rotational motion of the head.

To address some of these shortcomings and to distinguish between the performance of helmets that met the requirements of regulatory standards, some helmet rating methods have been developed.^11,24,59,105,108^ These methods include oblique impacts and use both linear and rotational kinematics of the head to evaluate helmet performance. The inclusion of both linear and rotational kinematics is guided by field knowledge of brain injury biomechanics.^54^ The testing protocols of rating methods are developed to best assess protection capabilities and performance of helmets in realistic impact scenarios, with parameter selections based on the best available knowledge of real-world impacts and injuries. Our knowledge of cyclist head impact conditions continues to develop with the emergence of new research. Advances to protective technology (such as better vehicle structure and new helmet technologies) and the introduction of new testing methodologies or standards contribute to changes in cyclist injury profiles. These factors, in addition to the ever-changing collision landscape, geographical variation of user groups and infrastructure lead to a requirement for regular review and revision as new data becomes available.

In order to rate helmets in a scientifically driven manner, we must set up a meaningful laboratory assessment which is comprehensive, and representative of the wide variety of scenarios with varied kinematics cyclists are exposed to in the real world. This includes selecting the impact location, speed and angle as well as choosing appropriate head injury criteria which relate to key cyclist head injury pathologies. The type of head injury can be obtained from a range of data sources including hospital data (neurosurgery, general admission, level 1 trauma and paediatric centres), road traffic collision data and fatality registers.^82^ Detailed physical or computational reconstruction of real-world collisions from scene evidence such as the damaged helmet or footage of the incident is the gold standard for determining injury mechanisms.^30,89^ Impact location is usually determined by assessing reclaimed cycle helmets which were involved in collisions.^104,118^ Impact speed and angle are generally extracted from reconstruction of head-vehicle or head-ground impacts, primarily modelled computationally using multibody simulation software such as MADYMO^13,14,17,30,95,132^ or alternatively with physical drop tower testing.^12,104,118,125^ Despite all these efforts, helmet testing methods to date often rely on data collected over 30 years ago in spite changes to vehicle fronts, infrastructure, and helmet technology. There is therefore a requirement for a contemporary review of the types of pathologies sustained by cyclists in real world collisions and physical characteristics relating to recent cyclist head impacts from real-world data, particularly for different injury types.

In this paper we review the available literature relating to cyclist head injuries and impact mechanisms from real-world collisions, computational reconstruction and laboratory testing. The literature included focuses on a variety of contributing risk factors such as cyclist characteristics (age, sex and intoxication level), collision kinematics (head impact locations, speeds and angles) and impact characteristics (helmet type and/or use and type of object impacted). We investigate different types of head injury pathology, including their prevalence, mechanism and the existing protective effects of helmets against them. As different types of head injury pathology have different mechanisms, we report in detail on key studies which investigate specific pathologies.^87^ This is particularly important for determining the existing capabilities of helmets and being able to understand how to differentiate between the protective effect of different helmet technologies against a range of head injury types.

### Literature Selection

This literature was selected from online databases (Google Scholar, PubMed, Scopus and Web of Science) as well as applying a snowball approach to assess the relevance of any references included in the selected literature.^116,119^ Papers were limited to those where an English translation could be obtained. We ensured that the most contemporary findings are included within this study. Once studies were identified, the abstracts were scanned in order to shortlist relevant studies for full review. More than 100 papers were identified for inclusion.

## Head Injury Type and Severity in Cyclists Involved in Road Traffic Collisions

**Figure 1:**
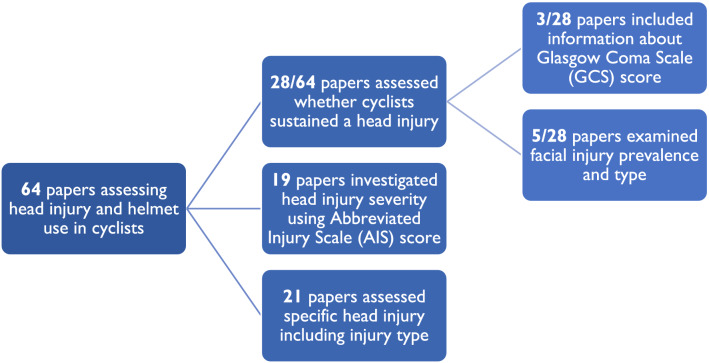
64 papers reviewed to examine head injury pathology, grouped by method of classifying head injury. The papers were stratified by head injury classification method in order to differentiate between different levels of detail relating to head injury. Nearly half of the papers included for review only assessed the binary metric of whether or not a head injury was sustained. There were some overlaps between the 19 papers which used AIS score and those which assessed head injury type including specific pathology.

The severity and types of head injury sustained by cyclists have been extensively investigated within the literature. We identified 64 papers which included information around head injury and helmet use in cyclists (Fig. 1). Of these, 28 papers reported on whether cyclists sustained a head injury or not, with three additionally including GCS (Glasgow Coma Score) and five additionally examining facial injury. A further 19 studies investigated head injury severity using AIS (Abbreviated Injury Scale) score. However, only 21 papers addressed the prevalence of one or more specific pathologies, either as a comparison to helmeted groups or not. Here we focus on these papers, as different pathologies have different injury mechanisms and therefore plausibly different preventative strategy.^41,51,52,54,90,126,127^ This is important in helmet testing, as there are many pathologies suffered by cyclists which are potentially mitigated in different ways. The performance of helmets to prevent a wide range of pathologies should be tested to assess comprehensive safety. The 21 papers which assessed specific head injury types (such as skull fracture, subarachnoid haemorrhage, subdural haematoma, extradural haemorrhage or diffuse axonal injury) involved approximately 90,000 cyclists. Many of these papers adopt the term traumatic brain injury (TBI) to group different types of intracranial injuries. Intracranial injuries are defined in the studies as lesions within the skull cavity, for example intracranial bleeding (including subarachnoid haemorrhage, subdural haematoma and intraventricular haemorrhage), focal lesions and diffuse axonal injury.^7,35,88^ One study by Sethi *et al*.^101^ additionally included skull fracture in their intracranial injury grouping.^101^ Other papers refer to mild TBI which includes symptoms such as post-traumatic amnesia, loss of consciousness or confusion. These studies are summarised in Tables 1 and 2 and reviewed in the subsequent sections.

**Table 1:** Summarises the studies reporting on head injury type in cyclist incidents, identified during the literature review process.

Data source	Study	Dataset	Data year(s)	Summary of key findings	Helmet information
General Hospital Admissions	Chiron et al.^19^	Rhône registry, France	1996	Mild problems of consciousness without neurological symptoms were most common (31%)	Unknown helmet use within study
	Amoros et al.^3^	Rhône registry, France	1998–2008	There were 488 (21%) AIS2 + head injuries, and 101 (4%) AIS2 + facial injuries. 69% (189 of 273) AIS4 + injuries were to the head	Unknown helmet use within study
	Ndiaye et al.^78^	Rhône registry, France	2005–2014	Extradural haematoma was common in cyclists compared to other users	Unknown helmet use within study
	Ganti et al.^36^	US emergency department admissions	Jan. 2008–Aug. 2010	Non-helmeted cyclists had higher prevalence of skull fracture, haemorrhages (extradural, subdural, subarachnoid, intraparenchymal and intraventricular) and facial fractures	29/143 (20%) wore a helmet
	Lindsay et al. *paediatric^61^	Paediatric admissions to 15 Canadian hospital emergency departments	2004–2009	Skull fracture and concussion were common in non-helmeted cyclists	Overall, 62.2% of cyclists wore helmets. For admissions, 54.1% wore a helmet. For those with head injury, 46.1% wore a helmet
	Baschera et al.^8^	Admissions to a hospital in Western Australia	2008–2015	187 sustained a TBI. Helmet use was associated with a reduced prevalence of extradural hematomas and open head injuries	Helmet status is given among the TBI cohort. 113/187 of cyclists with a TBI wore a helmet, while 59/187 had no helmet. 15/187 had unknown helmet status. The vast majority (88%) of cyclists with TBI were suffering from mild TBI
	Alfrey et al.^2^	Admissions to Marin County Level III Trauma Center in California, USA	2007–2015	In helmeted riders there were significantly fewer facial fractures 67/701, 9.5%, vs. 35/205, 17.0% (), skull fractures and serious head injuries	701 (77%) wore helmets and 205 (23%) did not wear helmets
	Woo et al. ^120^	Hospitalised recreational cyclists in Hong Kong	2015–2019	Helmets protected significantly against skull fracture, extradural haematoma and subdural haematoma regardless of injury mechanism, age or antiplatelet medication intake	361 (84.7%) cyclists were not wearing a helmet. Although 47% of cyclists had intracranial haemorrhage, only 15% wore a helmet
Major Trauma Centre / Intensive Care Unit Admissions	Forbes et al.^35^	St Mary’s Hospital London (UK) admissions	2011–2015	Helmet use protected against subdural haematoma, skull fracture (down 80%) and intracranial injury (down 80%)	97 known helmet status (27 with, 70 without)
	Sethi et al.^101^	One New York (USA) major trauma centre admissions	Feb. 2012–Aug. 2014	Helmeted cyclists were less likely to sustain skull fracture, subdural haematoma and other intracranial injuries than non-helmeted cyclists	Of 699 patients, 273 (39.1%) were wearing helmets. Among the 335 patients given a head CT, 110 (32.8%) were wearing helmets
	Liu et al.^62^	Taiwanese Level I trauma centre admissions	Jan. 2009–Dec. 2013	Incidence of both subdural haematoma and subarachnoid haemorrhage increased with age	60/669 wore a helmet (9%) and 606/669 (90.6%) did not wear a helmet
	De Guerre et al.^22^	One level-I trauma centre admissions in The Netherlands	2007–2017	Subarachnoid haemorrhage and subdural haematoma were common	Majority of cyclists were not wearing a helmet (1838, 92.5%) and the remaining 7.5% (148) wore a helmet
	Dodds et al.^29^	Trauma Audit Research Network (TARN) admissions to the majority of England and Wales major trauma centres	Mar. 2012–Sep. 2017	In non-helmeted cyclists, skull fracture (27% vault and 26% base), subarachnoid haemorrhage (24%), subdural haematoma and cerebral contusions (both 22%) were most common compared to subarachnoid haemorrhage (9%), cerebral contusions (8%) and base of skull fracture (7%) in helmeted cyclists	Of the 6,621 with known helmet status, 4,075 (61.5%) wore a helmet and 2,546 (38.5%) did not wear a helmet
	Carone et al. *paediatric^16^	Paediatric intensive care admissions at a UK hospital	2011–2018	Extradural haematoma, contusions and subdural haematoma were most common	17.3% of the population wore a helmet
Road Traffic Collision Databases	Bambach et al.^7^	New South Wales, Australia linked police-reported crash, hospital admission and mortality data	2001–2009	The more severe the head injury, the greater the risk reduction wearing a helmet provided, particularly for skull fractures, intracranial injuries and open head wounds	5087/6745 (75.4%) cyclists wore a helmet. Head injured cyclists were: less frequently wearing a helmet (44.1–58.4% compared with 77.2%)
	Leo et al.^57^	Swedish cyclists from the Swedish traffic accident data acquisition (STRADA) national road accident database and Dutch cyclists from the Dutch Institute for Road Safety Research collision database, SWOV	2016–2018	Cerebrum injuries, cerebral concussion with loss of consciousness for less than 1 h and base of skull fractures were most common	No helmet wearing rate in this study, though it is noted that helmet usage is higher in Sweden than in the Netherlands (according to national statistics)
	Baker et al.^4^	Casualties from Great Britain’s Road Accident In-Depth Studies (RAIDS) database	2013–2020	Subarachnoid haemorrhage and skull fracture were common pathologies. Wearing a helmet was associated with a lower prevalence of any severity TBI, mild-to-moderate severe TBI and skull fracture and subdural haematoma. Two helmeted cyclists and 12 non-helmeted cyclists sustained a skull fracture. No helmeted cyclists sustained an SDH, compared to 8 non-helmeted cyclists who did	A subset of 94 (84% of 112) cyclists had known helmet status. There was an almost exactly even split between those who wore a helmet (46, 49% of 94 cyclists) and those who did not (48, 51% of 94 cyclists)
Hospital (mixed, all-severity settings)	Feler^34^	Adult cyclists identified using ICD10 in the USA’s National Trauma Data Bank®	2017	Skull fracture and intracranial haemorrhage were common in cyclists, with skull fracture more prevalent in male cyclists	36.4% (6,768/16,181) of cyclists in this cohort wore a helmet
Neuro-surgical Admissions	Depreitere et al.^25^	Belgian neurosurgical admissions	1990–2000	Skull fractures were most prevalent (86%), followed by brain contusions (73%) and subdural haematoma (43%)	83/86 non-helmeted and 3/86 helmeted bicyclists
	Park et al.^88^	Cyclist admissions to a Korean neurosurgery department	2007–2016	Skull fracture was the most prevalent pathology followed by subdural haematoma, subarachnoid haemorrhage, intracerebral haemorrhage and haemorrhagic contusion. Subdural haematoma incidence increased with age, as did other intracranial bleeds	Helmet use not accurately recorded and therefore not included
Fatally Injured Cyclists	Piras et al.^94^	Fatalities from the Brescia institute, Lombardy region, Italy	1983–2012	Common injuries included skull base fractures (117), cranial vault fractures (116), facial skeleton fractures (37)	No helmeted cyclists included in the sample

We separate the studies by data source group, to highlight differences between findings. We provided details of the specific dataset and years of data, a summary of the key findings relating to injury type and any factors of influence from the study and report the proportion of helmet use. Quantitative values corresponding to the number of cyclists included in the study, the number who sustain skull fracture, subarachnoid haemorrhage, subdural haematoma, contusion to the brain, extradural haematoma, DAI and overall mild and severe TBI are included. Where possible, the values and percentages are reported separately for helmeted and non-helmeted users to provide a clearer picture of the differences in injuries between these more and less protected groups

**Table 2:** Quantitative values corresponding to the number of cyclists included in the study.

Study	Number of cyclists overall	Pathology				TBI severity			
		Skull fracture	Subarachnoid haemorrhage	Subdural haematoma	Contusion	Extradural haematoma	DAI	Mild TBI	Severe TBI
Chiron et al.^19^	1541 total	–	–	–	–	–	–	103 (6.7%)	40 (2.6%) *[AIS3* +*]*
Amoros et al.^3^	13,684 *[6230 AIS2* + *injuries] total*	34 (0.2%) *[AIS2-3]* 15 (0.1%) *[AIS4* +*]*	–	47 (0.3%) *[AIS4* +*]*	11 (0.1%) *[AIS4* +*]*	39 (0.3%) *[AIS4* +*]*	1 (0.0%) *[AIS4* +*]*	1192 *[AIS2-3 injuries]* (19.1% of 6230 AIS 2–3 injuries)	189 *[AIS4* + *injuries]* (3.0% of AIS2 + injuries, 69.2% of 273 AIS4 + injuries)
Ndiaye et al.^78^	4913 total	–	–	–	–	–	–	–	–
Ganti et al.^36^	478 total	81 (17%)	172 (36%)		112 (23%)	–	22 (5%)	360 (75%) *[GCS* > *13]*	96 (20%) [*GCS* < *13]*
Lindsay et al. *paediatric^61^	15,569 total	338 (includes facial) (2.2%)	–	–	–	–	–	2278 (8.6%)	166 (0.6%)
Baschera et al.^8^	1019 total	63 (6.2%)	36 (3.5%)	36 (3.5%)	–	10 (1.0%)	–	164 (16.1%)	23 (2.3%)
Alfrey et al.^2^	701 helmeted	8 (1.1%)	–	–	–	–	–	299 (42.6%)	6 (0.9%)
	205 non-helmeted	9 (4.4%)	–	–	–	–	–	86 (42.0%)	8 (3.9%)
	906 total	17 (1.9%)	–	–	–	–	–	385 (42.7%)	14 (1.6%)
Woo et al. ^120^	65 helmeted	7 (12.5%)	9 (16.1%)	4 (7.1%)	6 (10.7%)	0 (0.0%)	–	–	–
	361 non-helmeted	75 (25.3%)	71 (23.9%)	63 (21.1%)	40 (13.5%)	20 (6.7%)	–	–	–
	426 total	120 (28%)	126 (30%)	103 (24%)	81 (19%)	30 (7%)	-	375 (88%)	51 (12%)
Forbes et al.^35^	27 helmeted	4 (14.8%)	3 (11.1%)	2 (7.4%)	–	0 (0.0%)	–	–	7 (25.9%) intracranial injury
	70 non-helmeted	40 (57.1%)	22 (31.4%)	21 (30.0%)	–	8 (11.4%)	–	–	43 (61.4%) intracranial injury
	323 total	58 (20.0%)	31 (9.6%)	26 (8.0%)	32 (9.9%)	13 (4.0%)	–	–	129 (39.9%)
Sethi et al.^101^	110 helmeted cyclists who had a CT scan	7 (6.3%)	5 (4.5%)	0 (0.0%)	4 (3.6%)	0 (0.0%)	1 (0.9%)	–	7 (6.3%) any intracranial injury on CT
	225 non-helmeted cyclists who had a CT scan	44 (19.7%)	23 (10.3%)	18 (8.1%)	18 (8.1%)	13 (5.8%)	2 (0.9%)	–	44 (19.7%) any intracranial injury on CT
	699 total	35 (5.0%)	28 (4.0%)	18 (2.6%)	22 (3.1%)	13 (1.9%)	3 (0.4%)	–	52 (7.4%) *[AIS3* +*]*
Liu et al.^62^	699 total	36 (5.4%)	63 (9.4%)	65 (9.7%)	42 (6.3%)	22 (3.3%)	–	136 (19.5%)	53 (7.9%) *[GCS* < *13]*
De Guerre et al.^22^	1986 total	393 (19.8%)	338 (17.0%)	330 (16.6%)	–	107 (5.4%)	–	1346 (67.8%)	
Dodds et al.^29^	4075 helmeted	Vault: 238 (5.8%) Base: 308 (7.6%)	358 (8.8%)	240 (5.9%)	317 (7.8%)	77 (1.9%)	100 (2.5%)	–	780 (19.1%)
	2546 non-helmeted	Vault: 695 (27.3%) Base: 658 (25.8%)	614 (24.1%)	561 (22.0%)	553 (21.7%)	313 (12.3%)	93 (3.7%)	–	1211 (47.6%)
	6621 total	Vault: 933 (14.1%) Base: 966 (14.6%)	972 (14.7%)	801 (12.1%)	870 (13.1%)	390 (5.9%)	193 (2.9%)	–	1991 (30.1%)
Carone et al. *paediatric^16^	133 total	> 1 (> 0.8%)	–	4 (3.0%)	4 (3.0%)	6 (4.5%)	1 (0.8%)	3 (2.3%)	22 (16.5%)
Bambach et al.^7^	6745 total	118 (1.7%)	–	–	–	–	< 8 (< 0.1%)	257 (3.8%)	482 (3.7%)
Leo et al.^57^	15,500 total	Base: 748 (4.8%)	–	–	–	–	–	–	–
Baker et al.^4^	46 helmeted	2 (2%)	–	0 (0%)	–	–	–	–	–
	48 non-helmeted	12 (13%)	-	8 (9%)	–	–	–	–	–
	112 total	16 (14.3%)	26 (23.2%)	8 (7.1%)	-	4 (3.6%)	0 (0.0%)	29 (25.9%)	43 (38.4%)
Feler^34^	18,604 total	1873 (10.1%)	–	–	–	–	–	–	3341 (18.0%) *[AIS3* +*]* 1299 (7.2%) *[GCS* < *13]*
Depreitere et al.^26^	86 total	74 (86.0%)	45 (52.3%)	29 (33.7%)	63 (73.3%)	34 (39.5%)	11 (12.8%)	–	86 (100%)
Park et al.^88^	205 total	119 (58%)	90 (43.9%)	98 (47.8%)	–	42 (20.5%)	13 (6.3%)	147 (71.7%)	43 (21.0%)
Piras et al.^94^	335 total	Vault: 116 (34.6%) Base: 117 (34.9%)	–	–	–	–	–	–	197 (cause of death) (58.8%) 219 (lethally hit) (65.4%)

The asterisk (*) refers to studies which only included paediatric patients

The number who sustain skull fracture, subarachnoid haemorrhage, subdural haematoma, contusion to the brain, extradural haematoma, DAI and overall mild and severe TBI are included. Where possible, the values and percentages are reported separately for helmeted and non-helmeted users to provide a clearer picture of the differences in injuries between these more and less protected groups

### Challenges in Determining Common Pathologies in Bicycle Collisions Due to Data

At a high level, the literature identifies skull fracture, subdural haematoma and subarachnoid haemorrhage as being the most common severe TBI pathologies for cyclists.^4,16,26,29,34,36,22,61,72,77,94^ Mild injuries such as soft tissue injuries and short periods of loss of consciousness are prevalent in less severely injured cohorts. The data sources with sufficient data available for analysis frequently come from settings which treat and capture a high proportion of severe TBI. Therefore, information about mild TBI in cyclist populations is limited, leading this group to be under-represented. In addition, there is no consensus on the definition of mild TBI or its classification, leading to additional complexity.

**Figure 2:**
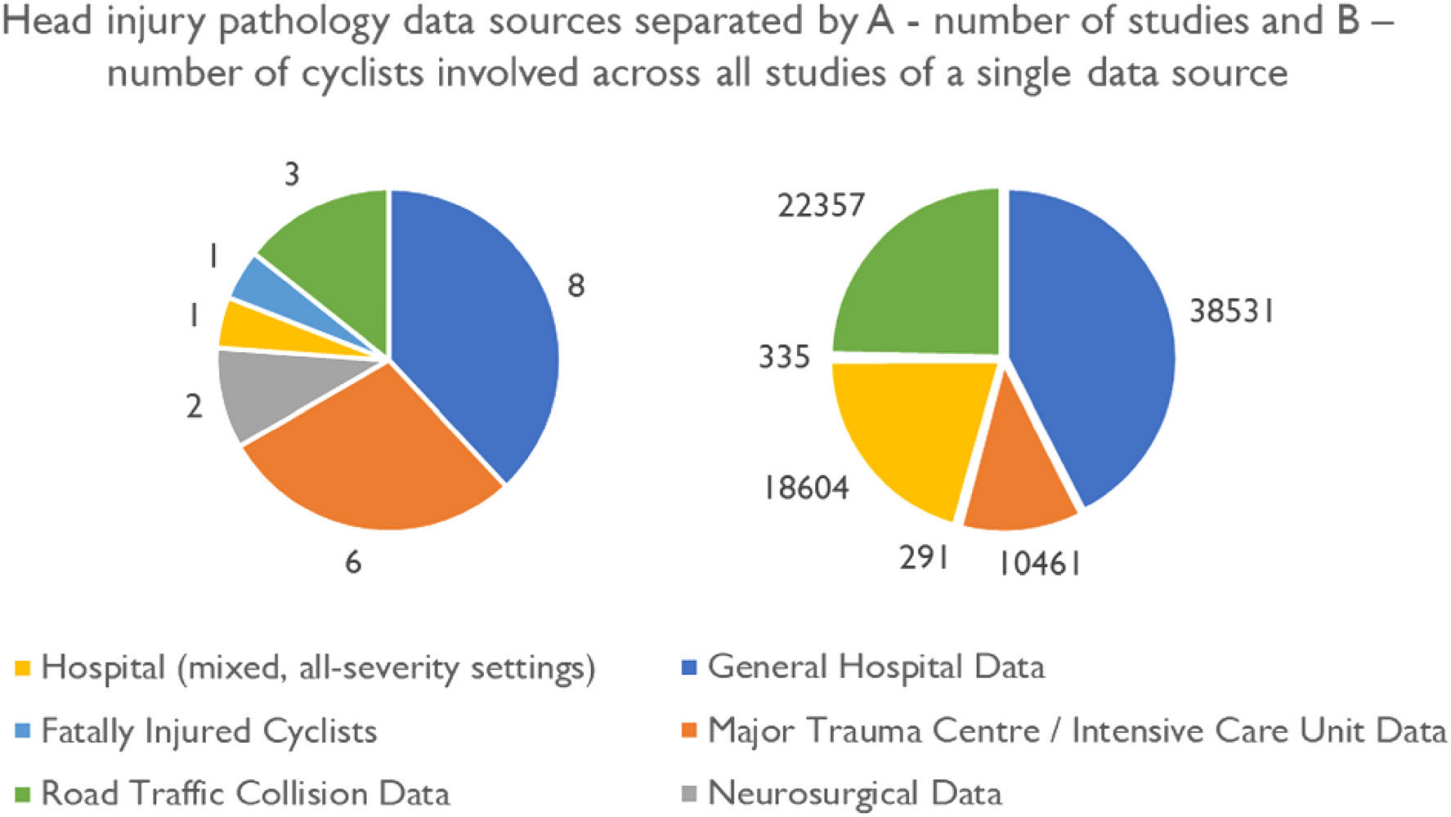
The proportion of different data sources that the head injury pathology literature reviewed relies on. The data source is relevant as it often relates to the overall severity of either the global or head injuries sustained by a cyclist. Both the number of studies and cyclists involved in those studies is captured.

However, due to different studies using different data types to draw conclusions, it is challenging to understand the precise prevalence rates of different types and severities of injuries overall. Common injuries highlighted by studies depend on the setting the bicyclist injury data was collected, as summarised as best as possible in Tables 1 and 2. For example, for studies which rely on general hospital data, common head injuries tend to be milder,^19,61,78^ whereas for intensive care or neurosurgery settings common head injuries tend to be more severe.^26,29,22,88,101^ Figure 2 shows the proportion of studies separated by data source, which shows that the largest number of reported cycle head injuries are based on general hospital admissions, followed by all-severity hospital settings and road traffic collision databases. This figure highlights the potential bias of the severity of injuries captured in previous studies. Hence, in the following sections, we review injury patterns based on each data source. We have summarised the numbers and percentages of each injury type sustained by cyclists from the different studies and data sources in Tables 1 and 2.

### Injury Patterns in General Hospital Admissions

Chiron *et al*.^19^ examined the head injuries sustained by 1541 cyclists who attended hospital in the Rhône region in the 1990s.^19^ They found that the majority of injuries were minor (45% were scalp injuries). Of the 333 with injuries to the cranium, 31% presented with symptoms associated with mild TBI (loss of consciousness without additional neurological symptoms) and 8% presented with symptoms associated with symptomatic TBI (dizziness and cephalalgia). Only 12% of the 333 cyclists with head injuries presented with AIS3 + severity head injury (including 2% who died). There were 15 cyclists with isolated head injuries, whose severe pathologies included three cyclists with extradural/epidural haematoma, two with basilar skull fractures, two with severe disruptions of the cranium and brain, and one with a contusion. Individual pathology rates were not reported for non-isolated head injuries. Amoros *et al*.^3^ later examined 13,684 cyclists from 1998 to 2008 Rhône registry data.^3^ 21% (488 of 2357) AIS2 + injuries were to the head and 4% (101 of 2357) were to the face. Of AIS4 + injuries, almost 70% (189 of 273) were to the head, with subdural and extradural haematoma most common, followed by skull fracture. The small number of AIS4 + facial injuries were maxilla fractures. A later paper by Ndiaye *et al*.^78^ examined 2005–2014 data from the same region found that extradural haematoma was particularly common in cyclists.^78^ Similar to Chiron *et al*., injuries were predominantly minor, including contusions and simple fractures. Lindsay *et al*.^61^ investigated paediatric head injury, including from cycling, in children admitted to Canadian hospitals from 2004 to 2009.^61^ The majority of injuries were minor.

Alfrey *et al*.^2^ explicitly explored less severe head injury. The authors report on cyclist injuries recorded by the Marin County Level III Trauma Center in California, USA.^2^ The sample included 906 patients collected from a general hospital during the full calendar years 2007–2015, of whom 701 (77%) wore helmets and 205 (23%) did not wear helmets. They include both severe and mild TBI within their analysis. Severe head injuries were split into skull fracture 17 (2%) and major head injury (14, 1.5%). For mild TBI, the authors adopt a symptom-based classification for their “concussion” group, which only includes casualties without any CT findings or intracranial bleeding. The list of symptoms included were: confusion and amnesia, repetitive questioning, memory loss, headache, and dizziness with or without nausea and vomiting. Similar proportions of mild TBI were found in both the helmet-wearing and non-helmet-wearing group. The authors acknowledge the subjectivity of the mild TBI finding due to a lack of a clear consensus on the definition of “concussion” and current challenges relating to diagnosing mild TBI using radiology. Sharp and Jenkins^102^ wrote an extensive review on the challenges surrounding the terminology of concussion, and the need to classify the severity of traumatic brain injury via a designated system.^102^ One such system is the Mayo classification system, whose mild and symptomatic categories overlap strongly with the list used by Alfrey *et al*.^2,66^. Sharp and Jenkins recommend that following TBI severity classification, the precise, underlying cause of post-traumatic symptoms are diagnosed if possible.

### Injury Patterns in Patients Requiring Neurosurgical Hospital Admission

A small study by Depreitere *et al*.^26^ examined in detail 86 injured (mainly non-helmeted, 83/86) cyclists presenting at hospital from 1990 to 2000.^26^ The data was taken from patients admitted to a neurosurgical department and therefore includes more severe head and brain injuries. There was a very high prevalence of skull fracture (86%) and cerebral contusions (73%), while subarachnoid haemorrhage, multiple or large contusions and subdural haematoma negatively impacted outcome. Park *et al*.^88^ studied 205 cyclists admitted to a hospital neurosurgery department from 2007 to 2016.^88^ Like other studies, they found that skull fracture was the most prevalent pathology followed by subdural haematoma, subarachnoid haemorrhage, intracerebral haemorrhage and haemorrhagic contusion. Two of the most severe pathologies, extradural haematoma and diffuse axonal injury occurred less commonly. Both Depreitere *et al*. and Park *et al*. found that age negatively impacted outcome within their respective neurosurgical patient cohorts.

### Injury Patterns in Those Admitted to Level 1 or Major Trauma Centres

Sethi *et al*.^101^ assessed cyclist injuries captured by a New York level 1 trauma centre between 2012 and 2014.^101^ They found that 40% of those admitted wore helmets. Helmeted cyclists underwent fewer head CTs and were less likely to sustain a head injury overall, particularly skull fracture, subdural haematoma and other intracranial injury. A study of around 2000 cyclists admitted to Netherlands major trauma centres from 2007 to 2017 found that subarachnoid haemorrhage and subdural haematoma were common.^22^ A large, particularly valuable study comprising over 11,000 cyclists from the UK’s Trauma Audit Research Network (TARN) database found differences in head injury between helmeted and non-helmeted cyclists.^29^ Skull fracture was the most prevalent head injury in non-helmeted cyclists (27% vault and 26% base) followed by subarachnoid haemorrhage (24%), subdural haematoma and cerebral contusions (both 22%). Helmeted cyclists had lower head injury rates, with subarachnoid haemorrhage (9%), cerebral contusions (8%) and base of skull fracture (7%) most common in helmeted cyclists.

### Injuries Sustained by Fatally Injured Cyclists

Piras *et al*.^94^ examined 335 fatal cyclist collisions from 1982 to 2012 and found that skull fracture, particularly basilar skull fracture, was very prevalent.^94^ A small study examined TBI in 22 paediatric cyclists. This is important, as children make up 25% of RTC fatalities and two thirds of those deaths are caused by TBI. Among the 22 paediatric cyclists included, the most common pathologies were extradural haematoma, contusions and subdural haematoma.^16^

### Road Traffic Collision Data and Reconstructions

Few studies assess head injury using road traffic collision data sources, likely due to the often more limited clinical information available. Nevertheless, Baker *et al*.^4^ investigated cyclists in Great Britain’s in-depth collision database, RAIDS.^4,25^ They found that subarachnoid haemorrhage and skull fracture were common head injury types in cyclists. Cyclist collision scenarios can also be examined from a biomechanical perspective. One large study published in 2019 recorded 15,500 cyclists in Sweden (2016–2018) and the Netherlands (2000–2014) found that the most common head injuries were serious cerebrum injuries, cerebral concussion with loss of consciousness for less than 1 h and base of skull fractures.^57^

### Older Age Negatively Impacts Injury Outcome

Age is likely to affect the head injury outcome in cyclists. However, there are only a few studies that have explored this. A study from Taiwan highlighted differences in pathologies among different aged cyclists. Liu *et al*.^62^ found that the incidence of both subdural haematoma and subarachnoid haemorrhage increased with age.^62^ Park *et al*.^88^ found that incidence of subdural haematoma and subarachnoid haemorrhage, increased with age, supporting Lui *et al*.’s findings from 2015, as well as reporting higher incidences of intracranial haemorrhage and haemorrhagic contusions.^88^ Depreitere *et al*.^26^ also found that older age negatively influenced outcome pertaining to head injury.^26^

### Helmets Reduce All Head Injury Pathologies But to Varying Degrees

Our review demonstrates that a wide range of pathologies are reduced with helmet use, to varying degrees. It is commonly reported that skull fracture and subdural haematoma are much less prevalent in helmeted cyclists, possibly due to their impact mechanism being associated with direct forces.^4,34,35^ Forbes *et al*.^35^ found that helmet use protected against subdural haematoma, skull fracture and intracranial injury, postulating that direct impact injuries are reduced.^35^ Feler^34^ similarly found that helmets reduced skull fracture.^34^ Ganti *et al*.^36^ assessed pathology differences in helmeted and non-helmeted recreational cyclists.^36^ Non-helmeted cyclists had higher prevalence of skull fracture, haemorrhages (extradural, subdural, subarachnoid, intraparenchymal and intraventricular) and facial fractures. Lindsay *et al*.^61^ investigated paediatric head injury and found that skull fracture occurred in non-helmeted children, and all but one child with a brain injury did not use a helmet.^61^ Woo *et al*.^120^ assessed TBI in hospitalised cyclists in Hong Kong.^120^ They also found that helmets protected significantly against skull fracture, extradural haematoma and subdural haematoma regardless of age, antiplatelet medication intake, or mechanism of injury. Baker *et al*. similarly found that wearing a helmet was associated with a lower prevalence of skull fracture and subdural haematoma.^4^ Within the UK’s TARN database skull fracture was most prevalent in non-helmeted cyclists (27% vault and 26% base) followed by subarachnoid haemorrhage (24%), subdural haematoma and cerebral contusions (both 22%).^29^ All pathologies were significantly reduced for helmeted cyclists, with subarachnoid haemorrhage (9%), cerebral contusions (8%) and base of skull fracture (7%) the most common pathologies. The greatest reduction was observed in skull fracture, with less marked reductions for other pathologies particularly diffuse axonal injuries. An Australian study found that helmet use was associated with a reduced prevalence of extradural haematomas and open head injuries.^8^ Although other pathologies such as subarachnoid haemorrhage and diffuse axonal injuries were reduced with helmet use, the difference was not as marked or as strongly reported. Therefore, when considering how helmets should be tested and rated in conjunction with specific pathologies, it is important to note these discrepancies.

Several studies have investigated whether helmets can prevent from injuries sustained at both the severe and milder end of the spectrum.^7,10,61^ Berg *et al*.^10^ reported on around 50,000 cycling related hospital admissions in the Swedish population from 1987 to 1996. The authors observed a reduction in both mild and serious head injuries (*e.g*. both concussion and skull fracture) among age groups where helmet use increased, which could not be attributed to any other factors.^10^ Bambach *et al*.^7^ specifically examined cyclists in collisions with motor vehicles from 2001 to 2009 in New South Wales, Australia.^7^ They found that the more severe the head injury, the greater the risk reduction wearing a helmet provided, particularly for skull fractures, intracranial injuries and open head wounds. Lindsay *et al*.^61^ included examination of more mild head injuries and found that concussion also occurred predominantly in non-helmeted cyclists.^61^ As can be seen, far fewer studies have explored the effects of helmets on milder head injuries, in part due to underreporting. There are several reasons for underreporting of mild TBI, from the high prevalence of bicycle only collisions resulting in no hospitalisation to the still-increasing diagnostic capabilities.^42,55^ Reporting of mild TBI pathologies is likely to improve as mild TBI diagnostic methods such as blood biomarker and saliva testing improve in sensitivity and are more widely implemented, as has been seen in other settings such as pitch-side in sport.^49,28^ Hence, further work, supported by these developments, is required to better understand the effect of helmets in preventing mild TBI.

### Current Standards Use Injury Metrics that Address Skull Fractures and Focal Injuries

Existing helmet designs, guided by standards, appear to be particularly mitigating against skull fracture and associated subdural pathologies. Peak linear acceleration (PLA) is linked to direct forces and has been shown to predict the risk of skull fracture and other focal injuries.^54,75,85^ Current bicycle helmet standards rely on examining the PLA during an impact. EN1078 for example requires helmets to ensure the PLA remains lower than 250 g during an impact at 5.42 m/s onto a flat anvil. Studies on injuries sustained in helmeted and non-helmeted cyclists have shown that skull fracture and associated pathologies have been reduced in helmeted cyclists.^35^ There are however pathologies that do not see such a significant reduction with current helmets, such as diffuse brain injury and intracranial haemorrhage. Metrics including peak rotational acceleration and BrIC (based on peak rotational velocity) have been shown to relate to diffuse axonal injury.^67,107^ Similarly, subdural haematoma (a sub-type of intracranial haemorrhage) has also been predicted primarily using rotational metrics, particularly rotational acceleration.^27,79,123^ Further details and literature thresholds are given in Appendix Table 5. We recommend that future helmet testing methods additionally test helmet performance against kinematic metrics associated with these pathologies.

### What Other Metrics Could be Used?

Diffuse head injuries and intracranial haemorrhage have been shown to relate to the rotational acceleration and velocity of the head during impacts.^39,63,64,84,83,89^ The risk of subdural haematoma, subarachnoid haemorrhage and other intracranial bleeding,^122^ as well as concussive effects^40,83,93,114,129^ and diffuse axonal injury (DAI)^21,40,53,129^ has been predicted using peak rotational velocity (PRV). Rotational velocity is used in the brain injury criterion (BrIC), which has been shown to relate to DAI.^107^ In addition, a wide range of pathologies have been associated with peak rotational acceleration (PRA), including subdural haematoma extensively.^27,31,63,64,79,84,123^ More details of kinematic metric thresholds and risk function values from the literature are summarised in Appendix Table 5. PRV, BrIC and PRA have significant associated literature and they can be considered for inclusion in future testing methods.

By including at least one rotational kinematic metric, future standards and ratings could ensure that the protective performance of cycle helmets against diffuse pathologies is tested. Additionally, including a rotational kinematic metric provides both a motivation and a framework for assessing future helmet technologies, particularly those which are designed to address head injury pathologies that are not as substantially reduced by current helmets.

Tissue-based metrics extracted from finite element (FE) models can also be used to predict brain injuries. Several FE models of the human head have been developed to predict the response of the brain tissue to head motion, measured with metrics such as maximum principal value of strain, strain rate, deviatoric stress or total stress tensors,^41,43,45,48,50,58,76,106,130,131^ or the response of axons and vessels, measured with axial strain or strain rate.^30,37,43,99,121,130^ Some studies have developed injury risk functions for FE model predictions, based on using sporting data classified as “concussion” vs. “no-concussion”.^9,53,113^ The consensus is that FE models can reasonably predict deformation of diffuse parts of the brain, thus more suitable for predicting diffuse brain injuries.^47^ Fahlstedt *et al*.^33^ compared results from different FE brain models together and with different kinematics metrics, in the context of cycle helmet oblique impacts.^33^ They showed good correlation between predicted brain strain (and strain rate) and rotational kinematics metrics, such as PRV, BrIC and PRA, in contrast to metrics based on linear kinematics of the head. Currently there is no universally adopted FE model. In addition, using FE models currently require expertise and computational resources. Moreover, there is good correlation between overall brain response predicted by FE models and head rotational kinematics. Hence, adoption of rotational kinematics metrics in future standards can be an important first step towards improving helmet standards without adding significant challenges.

### How Can Different Metrics be Used in Helmet Assessment?

Careful consideration around how additional kinematic metrics, such as PRV, are included in a rating method is necessary. It is important to maintain the inclusion of linear kinematic metrics which are the cornerstone of helmet assessments. The fact that linear kinematic metrics have underpinned helmet testing for decades and that focal injuries such as skull fracture and associated subdural haematoma see substantial, significant reduction in helmeted cyclists is noteworthy. This suggests that the inclusion of kinematic metrics known to relate to specific head injury pathologies has been influential in reducing the prevalence of these injuries in helmeted cohorts. The inclusion of linear metrics must remain to ensure that the great progress made towards mitigating focal head injuries does not stagnate. To better assess cycle helmet capability to mitigate from diffuse injuries associated with rotation, rotational kinematic metrics should additionally be tested for. Consideration around how to incorporate these additional metrics with existing linear metrics must be carefully considered.

For standard protocols this is simple: the same approach of having a maximum acceptable threshold can be applied. With a rating system, the purpose is not to determine whether a minimum acceptable safety threshold is passed, it is instead to provide a way of differentiating between products or technologies that are available. Therefore, depending on the goal of a given rating system, a weighting may be devised to enable both the linear and rotational components to be taken into consideration. Finally, to develop a rating method that encourages improvements in helmet design, the performance of helmets should be considered in selecting the injury risk calculation method and associated bands, and they should be reviewed periodically depending on the results.

## Head/Helmet Impact Location

Determining common impact locations of the head and helmet during bicycle collisions is important for setting up a feasible number of representative laboratory tests. Although any impact point is plausible, common collision kinematics is likely to lead to a higher incidence rate of certain impact points.^69^ The aim of this section is to determine regions which have higher impact incidence rates. We assess 10 studies which have examined head and helmet location from predominantly real-world impacts (the full summaries of these studies can be found in Appendix Table 6). In these studies, impact locations were determined from direct helmet damage, head soft tissue injury location, footage of collisions and computational biomechanics analysis of bicycle collisions from real-world data. In the Appendix Table 6, we additionally included one computational study, where the locations were determined from parametric variation of simulated, realistic real-world scenarios. Here, we limit our included studies to reconstructions of real-world collisions where the study or corresponding author was able to provide us with the location data required for meta-analysis.

### Meta-analysis of Impact Location

There are a number of papers which assess head impact location in cyclist collisions,^14,18,26,44,65,70,74,87,104,118^ collectively capturing 1809 impact locations to the head and helmet in real-world collisions, with a further 1792 facial impacts captured by Meng *et al*.^74^ Despite there being a substantial amount of available data, it is extremely challenging to collate the information due to differences in conventions adopted to define regions of the head/helmet. There are two overarching approaches: impact locations based on anatomical regions and on geometric regions.

**Figure 3:**
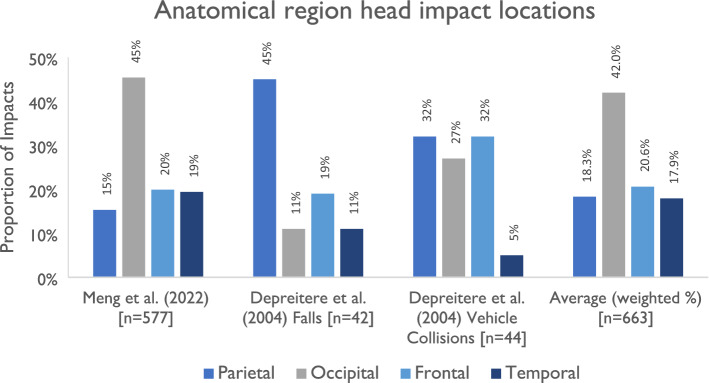
Shows the anatomical regions of head impact following cyclist collisions reported by Meng et al.^74^ and by Depreitere et al.,^26^ separated by collision type (bicycle-only or involving a motorised vehicle).^26,74^ The weighted average across the two studies (total *n* = 663) is also shown: 18% of impacts were to the parietal region, 42% were to the occipital region, 21% were to the frontal region and 18% were to the temporal region.

Two papers adopted regions based on anatomy. Depreitere *et al*.^27^ used head injury locations and corresponding anatomical regions based on skull bones.^26^ Meng *et al*.^74^ performed a similar analysis also including frontal, temporal, parietal and occipital skull regions.^74^ Depreitere *et al*.^27^ found that in bicycle-only collisions, 19% of impacts are frontal, 11% are temporal, 4% are frontotemporal, 45% are parietal and 11% are occipital (Fig. 3). In collisions involving a motor vehicle, 32% of impacts are frontal, 5% are temporal, 5% are frontotemporal, 32% are parietal and 27% are occipital. More recently, Meng *et al*.^74^ reviewed soft tissue injury impact location for a large cohort of 2039 cyclists from the German in-depth collision data, GIDAS. Among 3,115 soft tissue injuries, 577 were locatable to the head region. 19.9% of impacts are frontal, 19.4% are temporal, 15.3% are parietal and 45.4% are occipital.

As the remaining papers all assessed impact location via regions to the helmet or headform with geometric regional boundaries rather than anatomical, the remainder of this section focuses on geometric regions. There are two main approaches taken to define regions of the head/helmet, as shown in birds-eye view in Fig. 3. The first (approach A) involves separating the head/helmet into six 60° segments. The second (approach B) instead separates the head/helmet into four 90° segments. We group some of these segments together to form three main regions (front, side and rear) as well as the crown which corresponds to the top of the helmet. Note that the crown region is in the centre at the top of the head. There is not a consensus about the definition of the crown in terms of elevation angle, with Harlos *et al*.^44^ adopting the 60°–90° range.

**Figure 4:**
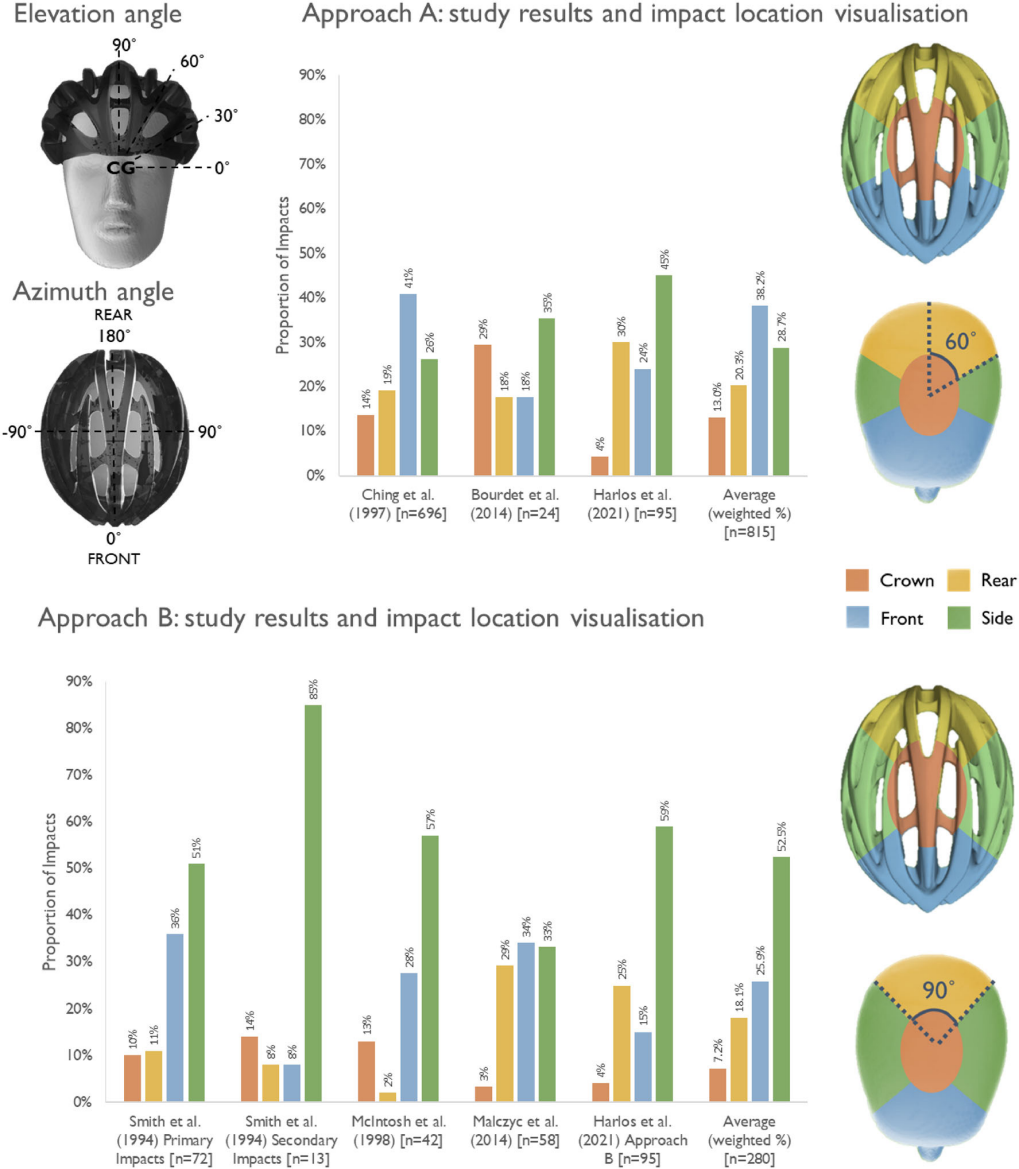
Shows the azimuth and elevation impact location method in addition to the two different main geometric approaches adopted in the literature and a summary of studies that have adopted approach A and approach B. Approach A is based on originally dividing the head/helmet into six 60° segments, which are then grouped to form regions: side (one left-hand and one right-hand segment), front and rear (two segments). Approach A is taken by Ching et al.^18^ and Bourdet et al.,^13^ while Harlos et al.^44^ can be mapped to any regions as the individual data points provided are based on elevation and azimuth angles. Approach B has four evenly distributed regions (90° segments), as seen by Malczyk et al.^65^ It was possible to map the data from Smith et al.^104^ and McIntosh et al.^70^ to the regions adopted in approach Harlos et al.^44^ can be mapped to any regions based on the raw azimuth and elevation angles being plotted within their paper. There is no clear consensus on the size of the crown region, however the proportional share to the top of the head tended to be small across all studies. The crown is illustrated as encompassing all 360° but only at the top of the head/helmet. The papers adopting approach A is given by the top panel, while the papers adopting approach B is given by the bottom panel. Separate averages are provided for each of the approaches. Harlos et al.^44^ provided sufficient raw data within the manuscript to use elevation and azimuth angles to perform analysis of two different regional boundaries and is therefore shown (with different regional boundaries) in the top and bottom plot.

**Figure 5:**
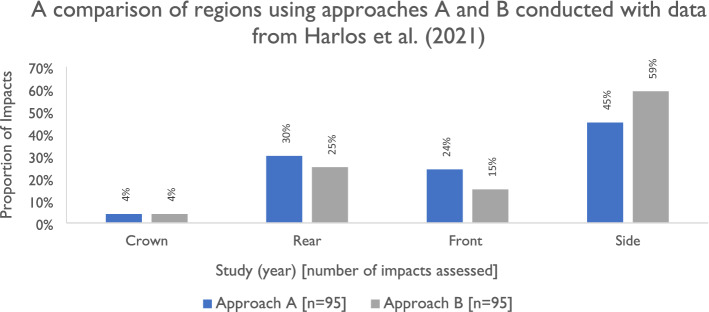
Shows a comparison of the regions in approach A and approach B created by mapping the Harlos et al.^44^ raw data points. In general, when a region is increased in size (*e.g*., the front/rear regions in approach A or the side region in approach B) the proportion of impacts which occur to that region also increases.

The reported values using approaches A and B are shown in Fig. 4. Two papers adopted approach A, including Ching *et al*.^18^ which reported the highest number of impacts. In approach A, the head/helmet is divided into six 60° segments, which are then grouped to form regions: side (one left-hand and one right-hand segment), front and rear (two segments). Four papers could be mapped to approach B, including Malczyk *et al*.^65^. This changes the relative size of the side, front and rear regions compared to approach A. In approach B we chose to assign one quarter to each of the front and rear, and two quarters to the side (one left, one right). The top region was assigned to the crown. Only one study gave impact locations in a way that enabled us to map their results to any regional boundaries. Harlos *et al*.^44^ mapped helmet impact locations in 3D computational space and subsequently measured azimuth and elevation angles relative to the centre of gravity of the NOCSAE headform which was used as a base. By providing the separate angles, we were able to perform a comparative analysis between the approaches A and B (Fig. 5). The vast majority of impacts occurred below 60° elevation (Fig. 4). Four impacts occurred above 60° at 64°, 74°, 75° and 81° elevation (three to the side, one to the rear) and are assigned to the crown (which is defined as 60°–90° elevation by the authors). We then analyse the azimuth angles of the 91 remaining impacts and apply both approach A and B to the dataset. Changing the region boundaries between the front, side and rear as shown above in.

Figure 5 produces different results in terms of the proportions of impacts to each region. In general, when a region is increased in size (*e.g*. the front/rear regions in approach A or the side region in approach B) the proportion of impacts which occur to that region also increases. There is a smaller difference in the proportion of rear impacts, but a more marked difference in the side and frontal impacts. 9 (10%) impacts occur between azimuth angles of 45°–60°, while 5 (13%) occur between azimuth angles of 120°–135°. The crown values are shown and remain unchanged as they are based on elevation rather than azimuth angles.

Two remaining studies reported helmet impact location results using groups described as “front”, “rear”, “side” and “top” but without any clear classification of the boundaries. Williams^118^ assessed 84 helmet impacts and found that 11% were to the crown, 7% to the rear, 16% to the front and 66% were to the side. Otte *et al*.^87^ assessed 82 impacts and found that 5% were to the crown, 7% to the rear, 24% to the front and 63% were to the side. We excluded one study from the meta-analysis due to the simulations not being based on real-world collisions. Bourdet *et al*.^13^ used approach A to assess simulated head impact location in 1024 cyclist falls from loss of control or kerb contact simulated in Madymo.^13^ They found that 6% of impacts were to the crown and the rear, 21% to the front and 73% were to the side.

There are several limitations and challenges relating to performing a meta-analysis of impact location data. We had to make an assumption to classify two studies as having used approach A or B based on their diagrams alone, as no angles were given in the text. Secondly, some studies related to impact location on the helmet while others related to impact location on the head. Incorporating data from both sources is very valuable, however aligning the regions defined for the head and helmet is challenging. In the absence of a standardised method for classifying impact location, we recommend that the robust approach of supplying individual datapoints using azimuth and elevation angles is adopted (Fig. 4), with a clear definition of the point that these angles are taken from, as seen by Harlos *et al*.^44^ and others Yu *et al.*^124^ This method has two key advantages. Firstly, the raw results can be classified by any regions that are deemed useful by those using the data. Secondly, the exact impact point can be extracted (as opposed to number of impacts within a specific region), which may have useful applications for studying impacts to particular regions of the head/helmet.

### Meta-analysis Shows that Side and Front Regions Are Impacted Most

Regardless of the selected approach (A or B), the front and side are the two most impacted regions (Fig. 4). In approach A, the size of the frontal region is larger and this is reflected in the highest share of impacts to this area (38%) on average for studies adopting or mapped to approach A. Contrastingly, in approach B the side regions are relatively larger, with the highest share of the impacts occuring to the side region (53%). For approach A, the weighted average of the percentage of impacts to each region is: crown (13%), rear (20%), front (38%) and sides (29%). For approach B, the weighted average of the percentage of impacts to each region is: crown (7%), rear (18%), front (26%) and sides (53%). It is important to mention that the large, recent study of soft tissue injury location performed by Meng *et al*.^74^ found a high proportion (45%) of occipital impacts (to the rear of the head), which is notably higher than in all other studies assessed.^74^ This difference could be driven by either the type of data captured by the German in-depth collision data used by Meng *et al*.^74^ or the use of soft tissue injury location directly to determine impact location, as many other studies rely on regions of visible helmet damage.

The rim is of particular importance as it is the dividing line between regions protected and not protected by the helmet. 25–50% of impacts occur near the rim line.^18,70^ This highlights the importance of testing the lower region of the helmet. Although helmets are not explicitly tested below the rim line in standards, a small sample have been shown to perform well when tested under these conditions. DeMarco *et al*.^23^ selected 13 commercially available bicycle helmets and performed drop tests with speeds ranging from 1 to 10 m/s at an impact point selected at/below the test line of most bicycle helmet standards.^23^ The authors found that 12 out of the 13 helmets passed the US Consumer Product Safety Commission (CPSC) standard with only one certified helmet with a PU liner not meeting the standard. Despite this positive result regarding the protection maintained for EPS liners for impacts below the standard test line, our recommendation for future rating and standard test methods is that the impact locations across the series of tests should represent the impact locations observed in real-world impacts, to best align assessment with real-world incidents. All four impact locations examined within this meta-analysis have a substantial portion of impacts, indicating that it is important to assess protection of all four regions. This location information could be applied to generate a weighting of the importance of laboratory helmet impact tests with different impact points in a rating scenario when determining an overall TBI prevention capability of helmets.

### Impacts to the Head Outside of the Regions Protected by a Typical Cycle Helmet

Areas outside of the helmet area are not well protected during an impact. Several of the studies within this section that examine Impact location report impact or injury to unprotected regions, such as the face, in helmeted users.^13,14,44,65,87^ Impacts to regions outside of the helmeted region is not generally reported in the location papers which extracted impact points from physically obtained helmets, as by definition this damage occurs to the helmeted regions.^18,70,104,118^ One study explicitly excluded the facial region.^26^ One study provided the breakdown of facial injury for 239 cyclists with injuries to the head region.^65^ 161 cyclists sustained injuries to the face, of which 94 were soft tissue injuries and 67 were skeletal. There were 140 cyclists with AIS1 facial injuries, 18 with AIS2 and 3 with AIS3. A second study assessed the differences in facial injury between helmeted and non-helmeted cyclists.^87^ The authors report almost no reduction in facial fractures among 433 helmeted cyclists (2.6%) and 3812 non-helmeted cyclists (2.7%). Although there was no significant effect observed (likely due to small sample size), a trend towards a protective effect of the helmet for the upper and mid facial regions was detected, with no difference observed for the lower facial region. The study which simulated cyclist impact scenarios found a higher proportion of facial impacts (approximately 5%) from skidding compared to kerb impacts (approximately 1%).^13^ The remaining studies provided less detailed information regarding facial impact. One study reported at least three instances of facial impact among 95 event descriptions (3%).^44^ Although not explicitly described, another paper provided tabulated data showing that 3 (limits: 1–5) [18%, limits: 6%-29%] impacts occurred outside of a general helmeted region.^14^ Another simply notes that there was at least one instance of facial fractures present within the cyclist cohort, with no further information given.^71^ One recent conference paper specifically assessed head impact location (including to the face) in cyclists using AIS1 + soft tissue injuries.^74^ The authors found that more AIS1 + soft tissue injuries were located in the face (64.8%) than the head (35.2%). The authors infer impact location from soft tissue damage, concluding that a high proportion of impacts occur below common helmet assessment lines, limiting the ability to assess full head protection. In summary, there is a need for additional future research to better determine the scenarios leading to facial impacts and injuries, as well as other commonly unprotected regions. A better understanding of the impact locations, rates and pathologies would enable researchers and designers to develop better helmet protection and test methods.

## Head Impact Speed

The impact speed of the head during a collision is important to inform the speeds to test helmets at so that they are representative of speeds experienced by riders in real world impacts. Unfortunately, only a small number of studies assess head impact speed in real-world collision scenarios. The scarceness of studies in this area is likely because estimating head impact speed from collision data requires expert facilities and knowledge to perform physical or computational reconstructions. Such reconstructions are not done routinely as part of collision data collection and coding. We identified two studies which use physical reconstructions and 5 studies which use computational reconstructions (4 real-world and one parametric). Table 3 summarises the seven identified studies which has estimated cyclist head impact speed, predominantly via computational or physical reconstruction. Further details are given in Appendix Table 7.

**Table 3:** Reported head impact speed in cyclist collisions with both vehicles and the ground, obtained by physical reconstruction of helmet impacts, physical crash test dummy testing with real bicycles and vehicles, computational reconstruction of real-world bicycle-passenger vehicle collisions and finally computational modelling of fall and loss of control scenarios.

Study	Method	Speed summary
Smith et al.^104^	Examination of 72 cycle helmets returned to the manufacturer following collision damage in New South Wales, Australia. The helmet damage was replicated to determine the impact speed	10 cases reconstructed gave 2.1–5.4 m/s head impact speed
Bourdet et al.^13^	Analysis of 1024 virtual head impacts obtained via parametric simulation of loss of control or kerb contact collision scenarios in MADYMO multibody simulation	For a cyclist travelling at 5.5 m/s, resultant velocities were found to be approximately 6.5 m/s (6.9 ± 1.2 m/s for skidding and 6.4 ± 0.9 m/s after hitting a kerb), normal component around 5.5 m/s (5.7 ± 1.3 m/s for skidding and 5.2 ± 1.0 m/s after hitting a kerb) and tangential 3.7 m/s (both skidding and falling). Normal components of the impact velocity were found to be approximately equal to the EN1078 test standard range (5.42 m/s)
Peng et al.^89^	In-depth collision data from 18 cases captured by Germany’s GIDAS database was selected for reconstruction using PC Crash and MADYMO	Head impact speed is lower than the vehicle impact speed The average of the head relative impact speed is 9 m/s (32.5 km/h) for bicyclist cases *Note that head impact speeds are overall higher in known vehicle-cyclist collisions than in cyclist only incidents (e.g., falls)*
Van Schijndel et al.^110^	Four physical crash tests replicating a collision between a Polar III ATD mounted to a bicycle (travelling at 15 km/h or static) and a Volvo V70 (travelling at 40 km/h) at a crossroad	Head impact speed ranged between 9.7 and 16.3 m/s, highest in the static rear impact. Three out of four impacts were with the A-pillar *Note that head impact speeds are overall higher in known vehicle-cyclist collisions than in cyclist only incidents (e.g., falls)*
Bourdet et al.^14^	Madymo simulation of 24 real-world cases involving cyclists with head injuries captured by French and German in-depth collision databases	Mean impact speed is 6.8 ± 2.7 m/s with 5.5 ± 3.0 m/s and 3.4 ± 2.1 m/s for normal and tangential components
Nie et al.^80^	Madymo simulation of 24 real-world cases involving cyclists with head impact sustained in collisions with vehicles in China	The ratio of the bicyclist's head relative impact speed to the vehicle impact speed was found to be 0.6–1.1. The average value of bicyclist's head relative impact speed was 33.9 km/h (9.4 m/s) *Note that head impact speeds are overall higher in known vehicle-cyclist collisions than in cyclist only incidents (e.g., falls)*
Verschueren^112^	MADYMO reconstruction of 22 cyclist collisions	Mean impact speed was 6.0–7.7 m/s

### Head Impact Speeds of Around 6-9 m/s are Common, but Higher Speeds Are Likely When Cyclists Impact Vehicles Travelling Above Typical Urban Speed Limits

**Figure 6:**
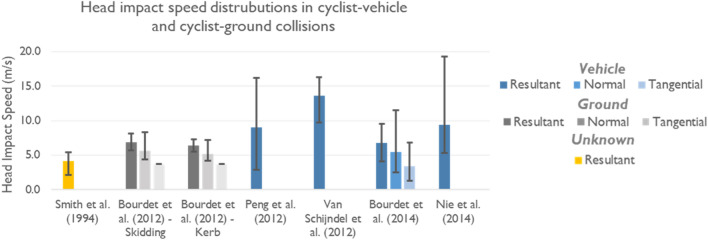
Shows the distribution of head impact speeds for cyclists involved in collisions. The blue tones indicate impact was with a vehicle while the grey tones indicate that impact was with the ground. Resultant head impact speed in m/s is shown for all studies. Where available, normal and tangential head impact velocities are additionally shown. The error bars show the data distributions, with the end of the bars indicating the maximum and minimum values. Data used to create this plot can be found in the Appendix.

Overall, average head impact speeds mainly range between 6.4 to 9.4 m/s, as shown in Fig. 6, with two outlier studies (4.1 m/s and a small study with 3 physically reconstructed vehicle impacts of 13.6 m/s). There is a concentration around 6.5 m/s for cyclist-ground head impacts and a higher concentration around 9 m/s for cyclist-vehicle impacts. The highest head impact speeds were associated with higher vehicle impact speeds.^80,89^ The three studies with normal components of the head impact velocity available ranged from 5.2 to 5.7 m/s (capturing both cyclist-ground and cyclist-vehicle collisions). These values are in agreement with the normal impact flat anvil testing speed of 5.42 m/s adopted by the EN1078 standard and is similar to other helmet testing methods.^32,59^

### Differences Between Collision Types

The analysis of the pathology data showed a relatively even split between cyclists who sustained head injuries in an impact with a motorised vehicle vs. in a cycle-only collision with no other vehicle involved. The papers reviewed which include vehicle involvement reported higher head impact speeds than those without vehicle impact.^14,80,89,110^ One study used returned physical helmets and did not provide information about whether there was vehicle involvement.^104^ Although there are 1024 simulated ground impact scenarios, no studies explicitly reconstructed real-world cyclist falls to assess head impact speed. Furthermore, we found only 69 vehicle-cyclist collisions that have been reconstructed to assess head impact speed. Cycle-vehicle collisions are likely to lead to higher impact speeds than falls. Literature reports that cyclist head impact speed tends to be slightly below vehicle travel speed.^89^ Vehicle impact speed has been shown to relate to injury outcome for vulnerable road users including cyclists.^4^ One study found that 70% of cyclist collisions occurred with vehicle impact speeds below 20 km/h.^89^ However, in several of the studies, there are reconstructions capturing instances of cyclists being contacted at speeds much higher than typical urban speed limits of 20–30mph (30-50 km/h, 9–13 m/s). Additional research into the head impact conditions of cyclists in real-world collisions would be of significant value. With the evidence currently available, future helmet assessment should consider the benefit of adding a higher impact speed testing condition to mimic both impact scenarios: cycle-motorised vehicle collision and cycle-only fall to the ground.

### Collision Data Source is Likely to Affect Head Impact Speed and Angle Calculations

The available data affects the types of scenarios that can be reconstructed and can influence the results. For example, reconstructions exist in the literature of scenarios where cyclists are impacted by vehicles travelling significantly faster than typical urban speed limits, despite the majority of cyclist collisions occurring in urban areas. In general, greater detail is collected in collisions which lead to more serious injuries, with some possible exceptions where CCTV or other footage is used. Therefore, it is likely that because these reconstructions often use an in-depth collision data source, they may be representing a severe subset of collisions leading to an underrepresentation of milder head injuries. Contrastingly, the speeds found by Smith *et al*.^104^ from helmets returned to the manufacturer by those involved in a collision, were much lower. In the instance an individual is well enough to return their cycle helmet, the collision is likely to be less severe. Therefore, there is a need for additional future research to help determine whether the higher speeds observed in the cyclist-vehicle collisions within this study are representative of all cyclist-vehicle collisions or due to the data source. The findings of such studies should be incorporated into future helmet rating and standards. In addition, it should be noted that the data available for reconstruction and the accuracy of reconstructions affect not only the speeds, but other head impact characteristics such as impact angle, which is explored in the subsequent sections.

## Head Impact Angle

We found five studies which investigated impact angle in detail. This small number is again likely due to the main method for extracting angle to be full computational reconstruction of collisions, which is a very data and time intensive process. Four studies reconstructed real-world collisions generally involving vehicles using multibody dynamics simulations. Real-world data was taken from in-depth collision information to determine the pre-impact scenario including speeds, angles and positions. The simulation was run to reproduce the kinematics of the collision, capturing the head impact angle. One further study created a virtual database again using multibody dynamics simulation, simulating single-cyclist 512 plausible loss of control and 512 plausible kerb impact scenarios using parametric variation. The head impact angle was determined in the simulated collisions. The head impact angles reported here are measured from the normal to the impact surface (Table 4).

**Table 4:** Reported head impact angle from multibody simulation in cyclist collisions (both bicycle-passenger vehicle and between the head and the ground in the cases of the falls).

Study	Method	Impact angle
Verschueren ^112^	MADYMO reconstruction of 22 cyclist collisions	Head impact angles ranged from 40° to 50°
Bourdet et al. ^13^	Analysis of 1024 virtual head impacts obtained via parametric simulation of loss of control or kerb contact collision scenarios in MADYMO multibody simulation	For cyclists travelling at 5.5 m/s, the impact angle is 35°. For 11.1 m/s, the angle is higher (57°)
Peng et al. ^91^	In-depth collision data from 18 cases captured by Germany’s GIDAS database was selected for reconstruction in using PC Crash and MADYMO	Impact angle ranged between 11° and 73°, with a mean value of 38.4°
Bourdet et al. ^14^	Madymo simulation of 24 real-world cases involving cyclists with head injuries captured by French and German in-depth collision databases	Generally, impacts occur at an average angle of 33° ± 20° to the normal 48% of impacts occur between 0° and 30° to the normal, 41% occur between 30° and 60° to the normal and only 11% occur at 60°–90° to the normal
Nie et al. ^80^	Madymo simulation of 24 real-world cases involving cyclists with head impact sustained in collisions with vehicles in China	With increased vehicle impact speed between 20 and 70 km/h, the head impact angle decreased from 83.5° to 10.9°, with a mean value of 45.7° measured from the normal

### Evidence of Oblique Head Impacts in Reconstructed and Simulated Cycle Collisions, with High Representation in the 30° to 60° Range

**Figure 7:**
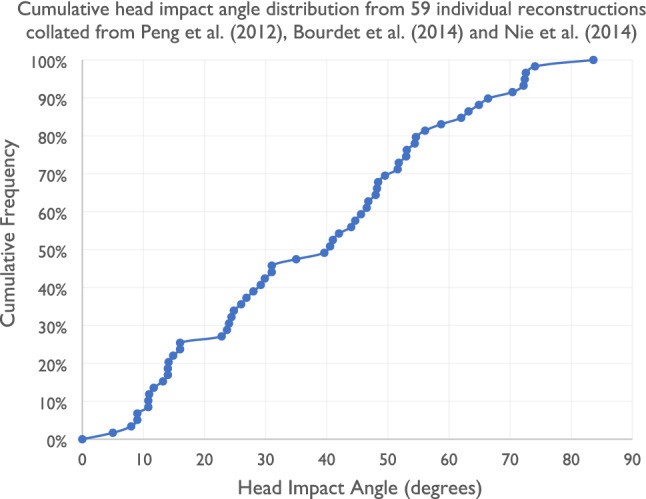
Shows the cumulative head impact angle distribution from 59 individual reconstructions collated from Peng et al.,^89^ Bourdet et al.^13^ and Nie et al.^80^ The 50th percentile from these three studies is just above 40°.

In general, the papers reviewed found that the angle to normal is most commonly between 0° and 60°, with averages of 35°, 33°, 38° and 46° quoted (Table 4). Figure 7 shows the cumulative head impact angle distribution from 59 individual reconstructions collated from Peng *et al*.,^89^ Bourdet *et al*.^14^ and Nie *et al*.^80^ The 50th percentile from these three studies is just above 40°.^14,80,89^ When considering falls from a standard cycle speed, angles in the region of 35°to 45° are common. In falls and skidding, it is reported that increased travel speed leads to increased angle of impact. For example, one study shows that the impact angle is increased from 35° at 5.5 m/s to 57° at 11.1 m/s,^13^ which is thought to be due to the increased tangential component present.^13,112^ Contrastingly, it is found that in collisions with vehicles, when cyclists were impacted at higher speeds, the head impact angle decreased from 83.5° to 10.9° (near-normal), with a mean value of 45.7°.^80^ As the angles range from highly oblique to near-normal across the range of vehicle impact speeds, there is value in both oblique and normal impact testing. Lower vehicle impact speeds are more common in urban areas where travel speeds tend to be restricted, therefore the inclusion of oblique impact testing in standards and rating methods has value to better represent impacts at lower travel speeds. In addition, the adoption of a 45° angle for oblique impacts in helmet assessment methods^60^ is supported by the heavy representation in the 30° to 60° range reported in the reviewed studies.

### Challenges Relating to Collision Reconstruction

When collisions are computationally reconstructed, a deterministic approach is often taken whereby the best-case scenario fitting the physical evidence is selected, as is the case for several reviewed studies.^13,14,80,89^ The accuracy of the reconstruction is then assessed by the authors of the studies in relation to any available physical evidence. One challenge with this approach is that small variations in input parameters can have a large effect on head impact parameters, particularly during the later phases of a collision. This can lead to a range of impact speeds and other kinematic metrics of the head such as force or acceleration for similar simulated scenarios. Uncertainty analysis is one approach that could be applied to determine the effects of the changes in input parameters on head impact characteristics and provide a confidence interval for the predictions.^6^

Another challenge is possible inconsistency in reconstruction methods between different studies. In fact, the majority of the computationally reconstructed cycle collisions are carried out by one research group. This is likely to ensure a high level of consistency among studies, but also demonstrates that the overall sample of studies reporting cycle collision reconstructions is small. This small number of simulated collisions with parameters available coupled with the ever-changing and geographically varied collision landscapes, protective equipment developments and changes associated to rider characteristics and behaviour results in a continued need to increase the number of publicly available reconstructions going forward.

Finally, there are also limitations relating to the tools adopted and available for computational reconstruction. For example, there are a range of multi-body dynamics ellipsoid, FE and hybrid facet (passive and active) human body models. The biofidelity of the musculoskeletal system and ability to react in a similar way to real-world vulnerable road users via active musculature vary for different models. The computational reconstruction studies which have assessed head impact speed and angle generally use Madymo with multi-body dynamics ellipsoid models, due to their rapid computational time that enables large numbers of simulations to be constructed.^13,14,80,89^ However, these widely adopted models have not been validated outside of the TB024-specified standard pedestrian dimensions. This results in a limited selection of available models (adult 5th percentile female and 50th and 95th percentile male), which does not capture large proportions of the population (particularly for the female sex). Increasing the range of scalable models and validation against cyclist scenarios will continue to strengthen the results relating to cyclist collision characteristics obtained via computational reconstructions.

## Impact Surface

The ratio between incidents involving and not involving a motor vehicle is dependent on the data source. As bicycle collisions involving motor vehicles tend to have more severe outcomes, they are more likely to be captured by datasets used for analysis in the literature.^94^ Within the papers reviewed for this study, there is an even split between impacts with a vehicle (*i.e.*, a motor vehicle collision) and with the ground (*i.e.*, a fall). However, among the 15 papers assessed for injury type amalgamated from different data sources, there was large study-by-study variation of the proportion of collisions involving motor vehicles from 4 to 100%. The overall weighted average proportion of collisions that involved a motor vehicle and that were bicycle-only falls were both 42% (details given in Appendix Table 8). It is reported that a high proportion (60–80%) of bicycle incidents do not involve another vehicle, and it is commonly accepted that these incidents are underrepresented in data.^3,98^ It is therefore likely that a number of ground-only impacts are unreported, and therefore underrepresented within this review compared to real-world prevalence.

### The Vast Majority of Cyclist Head Impacts are Against a Flat Surface

The proportion of impacts which occur on flat surfaces vs. edge-type surfaces is important as it can inform anvil selection for helmet testing. The purpose of this exploration is to determine the proportion of impacts which occur on flat surfaces vs. edge-type surfaces. Flat surfaces can be on the vehicle (*e.g*., windscreen) or the environment (*e.g*., road surface). Similarly, edge-type surfaces can be a road kerb or an edge structure of the vehicle (*e.g*., A-pillar). Only a small number of studies have assessed type of object contacted by the cyclist’s head during collision. Williams *et al*.^118^ assessed 72 helmets and found that all impacts occurred against flat surfaces. Smith *et al*.^104^ found that the majority of primary impacts occur on a flat surface (72%, *n* = 52) and only 3% occurred vs. a sharp object.^104^ More recently, Otte *et al*.^87^ performed extensive analysis of the GIDAS database involving 2844 cyclist impacts.^87^ They found that 88% of impacts occur vs. flat surfaces, a further 8% occur vs. edge structures of vehicles, with only 4% vs. edge structures in the surroundings, such as a kerb or post. Therefore, we can expect that assessing contact with flat surfaces will capture towards 90% of collision scenarios. As kerb stone impacts are adopted in the EN1078 European standards, this ensures a minimum performance threshold is already tested in helmets which are sold. Due to the fact that helmets undergo a pass-fail test for the potentially more severe kerb stone impact and the vast majority of scenarios are with flat surfaces, we recommend that flat rather than kerb stone anvils should be the focus for rating methods. Furthermore, while there are many different surface stiffnesses in real-world impact scenarios, rigid anvils with controlled coefficients of friction typically representing the road surface are adopted by standard and rating test methods. There is potential to increase the types of scenarios for testing helmets through adopting other surface stiffnesses.

### Coefficient of Friction

The impact surfaces can also have different coefficients of friction (*µ*). For example, vehicle surfaces are in general smoother than the road (*µ*_vehicle_: 0.2–0.3, *µ*_road_: 0.7–1.0).^5,103,115^ This can produce different responses of the head and helmet. In helmet testing, the coefficient of friction of the anvil has been found to significantly affect the helmet kinematics, in particular rotational motion.^92^ Current helmet standard and rating methods which include oblique impacts commonly use oblique anvil covered with 80-grid abrasive paper. This anvil condition represents an upper bound of the coefficient of friction. Further work is required to adopt suitable surface conditions that could better represent a range of impact media, such as road and vehicle surfaces.

## Conclusions and Future Directions

We reviewed literature to better understand the head collision characteristics of cyclists. We found that the key severe head injury types suffered by cyclists are skull fracture, subdural haematoma and subarachnoid haemorrhage. Mild injuries such as soft tissue head injuries and loss of consciousness are also common. However, one of the key challenges around identifying key head injury pathologies is that the data sources are biased towards either mild or severe head injuries, and they do not capture the full range of injuries from slight to fatal. Large studies focused on capturing all severities of injuries sustained by cyclists from uninjured to fatal including the prevalence of injury and pathologies is needed. These studies would provide valuable insight for researchers and designers developing better test methods and helmets, and those more broadly working in improving cycle safety and accessibility.

Wearing a helmet significantly and substantially reduces focal injuries like skull fracture and associated subdural haematoma prevalence. It also significantly reduces the prevalence of other TBI pathologies (*e.g*., different types of intracranial haemorrhage, cerebral contusion) but less substantially. Causally or coincidentally, there has been a historical focus of standards on reducing peak linear acceleration, which is well correlated with skull fracture, associated subdural haematoma and other focal injuries. We recommend that in addition to assessing linear metrics, rotational metrics such as peak rotational velocity and acceleration are included in future rating and testing methods. This will provide better insight into how helmets protect against diffuse injuries. In conjunction with this laboratory-based research, further investigation into how existing technology impacts diffuse injury outcome in real-world collision scenarios is required.

Our meta-analysis of 10 papers reporting 1273 impact locations showed that the majority of impacts occur to the sides (37%) and front (33%) of the helmet or head. Another common theme across much of the literature was the high proportion of impacts which occur near the base of the helmets along the rim line. It is important that these lower regions of the helmet are considered when formulating testing protocols.

Overall, average head impact speeds range between 4 and 13 m/s, with the majority of studies reviewed in the 6–9 m/s range. Current assessment methods generally include impact speeds in the 4.5–7.5 m/s range. The higher the travel and vehicle impact velocity, the higher the head impact velocity (and often more severe the injury). In most of the epidemiology data there is a relatively even split between cyclists with head injuries sustained when there is contact with a vehicle and in falls. Therefore, there may be a benefit of a higher impact speed testing condition to mimic the cyclist-vehicle impact scenario. More research is needed to understand the effect of cyclist-vehicle impact scenarios from different speeds and angles. This would enable testing standards and rating systems to better incorporate this impact scenario.

The angle of the impact to the normal is commonly less than 60°, concentrated in the 30°-60° range. This further justifies the use of oblique impacts. However, the studies leading to these conclusions are a few and future work is needed to provide more data on the distribution of head impact angles in cycle collisions.

The overwhelming majority of head impacts were found to occur vs. flat surfaces, either part of the vehicle or the ground. There were a much smaller proportion (below 10%) of incidences where the impacting object was edge-type. The kerbstone anvil is already included in standards testing required before helmets are sold in the market, which ensures a minimum edge-type impact protection threshold has been passed. Hence we suggest that additional rating systems focus on the flat surface impact scenarios which account for approximately 90% of scenarios.

The findings of this review can guide the improvement of existing cycle helmet assessment methods and development of new methods that better represent the head impacts of cyclists during real-world collisions and their different head injury types. This review also highlights several gaps in our current understanding and data, thus highlighting the urgent need for more research and better data with an aim to improve head protection in cyclist collisions.

## Appendix

See Tables 5, 6, 7 and 8.

**Table 5:** Kinematic injury thresholds, separated by head injury pathology or severity.

Pathology	Threshold	Evidence
Subdural Haematoma (SDH)	*PRA 4.5krad/s*^*2*^	Ommaya et al.^84^, Löwenhielm^63,64^
	*PRV 50-70 rad/s*	Ommaya and Hirsch^83^
	HIC 1429	50% risk assessed via a linear kinematic metric, Marjoux et al.^68^
	PRA 10krad/s^2^	The PRA of is an asymptote value for A lower value of 10 krad/s^2^ is suggested for GAMBIT. This threshold is further supported by PMHS experiments of Depreitere et al.^26^, which suggests a 10 krad/s^2^ threshold for bridging veins rupture (leading to SDH) when the pulse duration is shorter than 10 ms^27^. Interestingly, the new ECE22.06 regulation uses a 10.4 krad/s^2^ threshold for PRA in oblique impacts^31^. Also supported by Yoganandan et al.^123^
Skull fracture	*HIC 1000* *HIC 1500* PTA 180 g PTA 250 g PTA 120 g PTA 155 g HIC 667 HIC_36_ 1050 HIC_36_ 1680 HIC_36_ 2113 HIC_36_ 2351 PTA 135 g HIC 667	*16% risk life threatening SF (AIS4* +*)* Prasad and Mertz^96^ *56% risk life threatening SF (AIS4* +*)* Prasad and Mertz^96^ 5% risk Mertz et al.^75^ 40% risk Mertz et al.^75^ 15% risk – Vorst et al.^111^ 50% risk – Vorst et al.^111^ 5% risk – Horgan^46^ 50% risk of skull fractures at AIS2 + Kuppa^56^ 50% risk of skull fractures at AIS3 + Kuppa^56^ 50% risk of skull fractures at AIS4 + Kuppa^56^ The highest HIC with no skull fracture Prasad and Mertz^96^ 50% risk of skull fracture Peng et al.^90^ 50% risk of skull fracture from considering windscreen impact only (ignoring ground impact)—Marjoux et al.^68^
Diffuse Axonal Injury (DAI)	PRA 16krad/s^2^ BrIC 0.78	Moderate to severe DAI Margulies and Thibault^67^ 40% risk of AIS3 DAI Takhounts et al.^107^
Subarachnoid Haemorrhage (SAH)	–	–
Focal Injury	–	–
Mild Traumatic Brain Injury (mTBI)	PTA 66 g PRA 4.6krad/s^2^ PTA 82 g PRA 5.9krad/s^2^ PTA 106 g PRA 7.9krad/s^2^	25% risk of mTBI Zhang et al.^128^ 25% risk of mTBI Zhang et al.^128^ 50% risk of mTBI Zhang et al.^128^ 50% risk of mTBI Zhang et al.^128^ 80% risk of mTBI Zhang et al.^128^ 80% risk of mTBI Zhang et al.^128^
AIS2	BrIC 0.53 11.4krad/s^2^	50% risk of AIS2 Takhounts et al.^107^ 50% risk—Peng et al.^89^
AIS3 +	BrIC 0.87 PTA 200 g PTA 162 g HIC 1442 18,775 krad/s^2^	50% risk of AIS3 Takhounts et al.^107^ 50% risk of AIS3 + Richter et al.^97^ 50% risk of AIS3 + Peng et al.^90^ 50% risk of AIS3 + Peng et al.^90^ 50% risk of AIS3 + Peng et al.^90^
AIS4 +	PRA 16krad/s^2^ PRV 46.5 rad/s BrIC 1.06 PTA 250 g	Margulies and Thibault,^67^ Margulies and Thibault,^67^ 50% risk—Takhounts et al.^107^ 50% risk—Newman, 1986^79^
AIS4 + over 60 s (5 most common pathologies: SAH, SDH, skull fractures, soft tissue haematoma, scalp laceration)	PTA 203 g HIC 1082 12,753 rad/s^2^ 27.8 rad/s	50% risk of AIS4 + Wu et al.^122^ 50% risk of AIS4 + Wu et al.^122^ 50% risk of AIS4 + Wu et al.^122^ 50% risk of AIS4 + Wu et al.^122^
“Neurological injury”	CSDM 0.15	Marjoux et al. ^68^

**Table 6:** Reported head and helmet impact locations in cyclist collisions.

Study	Source	Impact locations
Smith et al.^104^	Examination of 72 cycle helmets returned to a US manufacturer following collision damage	*Primary impacts [**n** = 72]* Crown: 10% Rear: 11% Front: 36% Side: 51% *Secondary impacts [**n** = 13]* Crown: 14% Rear: 8% Front: 8% Side: 85%
Harlos et al.^44^	Examination of 95 cycle helmets returned to a US manufacturer following collision damage	*Approach A* Crown: 4% Rear: 30% Front: 24% Side: 45% *Approach B* Crown: 4% Rear: 25% Front: 15% Side: 59%
Williams^118^	432 casualties appearing at 11 hospitals in Victoria, Australia (1987–1989), examining 64 damaged helmets	63% of impacts occurred on the lower part of the helmet. Of those, 53% were fronto-temporal, compared to 44% fronto-temporal overall. 16% of helmets displayed evidence of multiple impacts. 11% (10) crown 66% (58) side 16% (14) front 7% (6) rear
Bourdet et al.^13^	Analysis of 1024 virtual head impacts obtained via parametric simulation of loss of control or kerb contact collision scenarios in MADYMO multibody simulation	6% crown (latitude 1) 73% side (longitude 2–5) 21% front (longitude 1) 6% rear (longitude 6) *Note-percentages here do not sum to 100% as the crown values are also captured in the side, front and rear regions. For this reason and the fact that the collisions are created via parameter sweep rather than real world reconstructions, we exclude this study from the meta-analysis*
Ching et al.^18^	311 helmets involved in 696 impacts were collected and analysed from cyclists with a head injury or helmet damage admitted to seven hospitals in Seattle (USA)	14% crown 26% side 41% front 19% rear Half (49.3%) of all impacts occur at the helmet rim.
Serre et al.^100^	Review of 862 damaged cycle helmets involved in 999 impacts in France	19% crown (*note: counted again in other regions*) 68% side (no significant difference between L and R side) 12% front 20% rear 46% of impacts occur to the lower region, these were also higher severity Small helmets were more severely damaged compared to large helmets *Note: the raw data was not available to determine the distinct distribution of the four regions, so this paper was excluded from the meta-analysis.*
McIntosh et al.^70^	Review of 24 damaged helmets involved in 98 impacts from collisions with vehicles or the road surface in New South Wales, Australia	13% crown 57% side 28% front 2% rear Helmet impacts tended to be close to the rim anterio-laterally, particularly for non-fatal (AIS ≥ 2) head injury to the temporal/parietal region.
Bourdet et al.^14^	Madymo simulation of 17 real-world cases involving cyclists with head injuries and known helmet damage captured by French and German in-depth collision databases	29% crown 35% side 18% front 18% rear 35% + impacts occur to the top, parietal region
Depreitere et al.^26^	Assessing impact point from soft tissue injury location of 49 cyclists (23 who fell, 26 involved with motor vehicles) admitted for neurosurgery at the University Hospital of Leuven, Belgium	The regions defined in the original paper were: frontal (29% for falls, 32% for collisions), temporal (11% for falls, 4% for collisions), fronto-temporal (4% for falls, 5% for collisions), parietal (45% for falls, 32% for collisions), occipital (11% for falls, 27% for collisions).
Malczyk et al.^65^	Examining dents and scratches on 26 helmets (58 impacts) from cyclists in Germany with clearly located soft tissue head injuries	3% crown 33% side 34% front 29% rear Non-helmeted cyclists more frequently injured temporoparietal and eye regions Helmeted cyclists sustained higher rates of frontal region impacts The top region displayed no injuries in either helmeted or non-helmeted cyclists. The regions used in the original text were defined as: frontal 16 (20.8)%, lateral 19 (24.7)%, Lateral Left 13 (16.9)%, Lateral Right 6 (7.8)%, vertex 10 (13.0)% and occipital 13 (16.9)%.
Otte et al.^87^	Examining helmet impact location from photographs (including visual severity assessment) from 82 collisions in Europe	5% crown 63% side 24% front 7% rear Lateral regions frequently are damaged. Left side suffered more damage than the right side. Where no head injury was sustained, there was damage to the top of the helmet (infrequent scenario). The regions used in the original text were defined as: frontal (24.4%), lateral (63.4%, 35.4% left and 28.0% right), vertex (7.3%) and occipital (4.9%).
McIntosh and Dowdell^71^	Examining primary impact site of 42 helmets damaged in Australia *This data was reused in McIntosh *et al*.*^70^* and therefore this study is not included in the main text*	13% crown 56% side 27% front 4% rear Two thirds of primary impacts occur to the frontal/temporal region. The regions used in the original text were defined as: frontal (27%), lateral (56%), vertex (13%) and occipital (4%).
Oikawa et al.^81^	Damage to 134 cycle helmets from Japan were categorised (crack/break/ deformation / abrasion)	Frontal and lateral regions of helmets are most frequently damaged (location data based on paper by Omada and Konosu.^86^ *Note that the raw data was unavailable and there was no translation available for the paper (which is written in Japanese) therefore this study was excluded from the meta-analysis *^86^

**Table 7:** Head impact speed data summary.

Study	Smith et al.^104^	Bourdet et al.^13^—Skidding	Bourdet et al.^13^—Kerb	Peng et al.^89^	Van Schijndel et al.^110^	Bourdet et al.^14^	Nie et al.^80^
Number of cyclists	10	512	512	18	3	24	24
Impact partner	Vehicle	Ground	Ground	Vehicle	Vehicle	Vehicle	Vehicle
Reconstruction method	Physical	Computational	Computational	Computational	Physical	Computational	Computational
Resultant velocity (m/s)	4.1	6.9	6.4	9.0	13.6	6.8	9.4
Smallest resultant velocity (m/s)	2.1	5.7	5.5	2.9	9.7	4.1	5.3
Largest resultant velocity (m/s)	5.4	8.1	7.3	16.2	16.3	9.5	19.3
Normal velocity (m/s)	–	5.7	5.2	–	–	5.5	–
Smallest normal velocity (m/s)	–	4.4	4.2	–	–	2.5	–
Largest normal velocity (m/s)	–	7.0	6.2	–	–	8.5	–
Tangential velocity (m/s)	–	3.7	3.7	–	–	3.4	–
Smallest tangential velocity (m/s)	–	–	–	–	–	1.3	–
Largest tangential velocity (m/s)	–	–	–	–	–	5.5	–

**Table 8:** Collision type for injury pathology studies with available information.

Paper	Collision types	Total No.	No. MVC	% MVC	No. Fall	% Fall
Chiron et al.^19^		1541	324	21.0	1143	74.2
Depreitere et al.^90^	44/86 accidents were collisions with a motor vehicle and 42/86 accidents were fall	86	44	51.1	42	48.9
Amoros et al.^3^	Collision type varies with cycling type: for MVC it is 8% for injured children, 17% for teenagers and adults injured outside towns and up to 31% for those injured in towns. The proportion of bicycle-only crashes varied with cyclist type, from 62.7% for 'teenagers and adults injured in towns', to 73.9% for those 'injured outside towns' and 85.4% for injured children	13,684	3113	22.8	9641	70.5
Bambach et al.^7^	Only MVC included	6745	6745	100.0	–	–
Lindsay et al.^61^	5.9% MVC, falls 82–91%	26,421	1559	5.9	22,854	86.5
Sethi et al.^101^	111 no helmet (26.5%), 77 helmet (28.4%), 188/699 overall, 64% of bicycle-related admissions were due to collisions with motorized vehicles, excluding 25 (6%) non helmeted and 32 (11.8%) helmeted incidents vs. pedestrian, rest falls (I think)	699	447	64.0	–	–
Liu et al.^62^	57.8% incidents from falls or loss of control, 21.5% involved a motorcycle, 13.3% involved a car, and 1.6% involved a truck/bus	699	254	36.4	404	57.8
Piras et al.^36^	Most cases involved a vehicle (92.84%). All vehicles: Car: 253, HGV: 40, motorcycle: 15, bike: 2; 3-wheel: 1. Motor vehicle involvement was the most frequent cause of fatal injuries. Falls only: 24 (7.16%)	335	311	92.8	24	7.2
Forbes et al.^35^	Motorized vehicle 63 (48.7%)	129	63	48.7	43	33.0
Park et al.^88^	Fall alone (46.8%), MVC (42.0%), Obstacle (11.2%)	205	86	42.0	96	46.8
Leo et al.^57^	Only includes cyclist-car collisions	15,650	15,650	100.0	–	–
Carone et al.^16^	15/22 vehicle, 6/22 falls, 1/22 obstacle	22	15	68.2	6	27.3
Baschera et al.^8^	More than half of cases sustained their injuries in a self-accident and about 40% in a collision with another vehicle, mainly in collision with cars and trucks	1019	408	40.0	510	50.0
Feler^34^	MVC: 7,196/16,181 (38.7%), information about falls not given	16,181	6262	38.7	–	–
Woo et al.^120^	4.0% MVC, 66.6% falls. Among the mTBI cohort: 12/353 involved MVs	426	17	4.0	284	66.7
Total	–	83,842	35,298	42.1	35,046	41.8
